# Crude Teachings

**Published:** 1871-09

**Authors:** 


### Editorial.

### CRUDE TEACHINGS.

It is unfortunate when a man who can teach some things fancies he can teach everything. When he has reached this sad state he is usually positive in direct proportion to his ignorance; and, as sound is often mistaken for sense, the most absurd statements are often accepted as scientific facts. Such a man may be honest and sincere, and yet by false teachings, do immense harm, especially if he is cordial and enthusiastic.

We have been led to these remarks by perusing about a page of printed matter, purporting to be the remarks of a friend we highly esteem, who has done much good to our profession, but who has also done much harm by presuming to teach where he has not yet learned. A quarter of a century will not relieve our profession from the evil results of the false teachings of this one individual, who might be useful, and only useful, if content to teach only that which he has studied, or, that he at least understands.

In a late issue we find this *teacher* saying, "The diamond and enamel may both be burned, leaving us their chemical elements," etc. A natural inquiry is, what are the elements of the diamond, and when we know that it is composed of the single element, carbon, and that the sole result of its combustion is carbonic acid, we naturally conclude that the teacher failed to know, or totally failed to impart his knowledge.

With the next breath he tells us, "There is no stone that is not an oxyd of some simple substance." The diamond is an oxyd of what? The beautiful stone called, fluorspar, is *scarcely* an oxyd. Did he forget the fluorides entirely? or had he failed to hear of them? To illustrate that every stone is an oxyd of "some simple substance," he mentions feldspar, which is composed of at least *five* simple substances besides oxygen.

The above is as good as the average of our friend's teaching. In the few sentences, here and there, which can be understood, ither by the speaker or hearer, we find such errors and misstatements the rule, and not the exception. They are asserted with the assurance of inspiration; and if any one ventures to doubt, he is told that the speaker is "going to vindicate the great Architect of the Universe," that "all things differ but in degree," "opthalmazoa," "crystalosophy, cellosophy, corpusculosophy," "differentiated." Of course the doubter pretends to be convinced, fearing to manifest the slightest shade of unbelief, lest the speaker should "marry his intellect and his affections," and in "spontaneity of natural production," the offspring should be a specimen of that "pragmatic pathology" which "includes all the progresses and regresses in the constructions and destructions of the individual (separate) domains of crystalosophy, cellosophy, and corpusculosophy, an exhaustive observation of whose processes will bring within the pale of consciousness all the possibilities of matter and mind."

We have written this much about a friend, and have written it as a friend. We know it will be misinterpreted, and our motives will be impeached, as usual, in some quarters. But we can not afford to be unfaithful to the whole profession, even at the dictates of private friendship. Nor can our profession in America afford to have such a medley of ideas, beclouded by such verbosity, pass for its advanced teachings. W.

